# Does operating theatre humidity affect the risk of surgical site infection? A review of the literature from 2005 to 2025^☆^

**DOI:** 10.1016/j.infpip.2026.100550

**Published:** 2026-05-05

**Authors:** Oluwadamilola Fasan, Ogheneochuko Andrew Saba, Lorna Gordon, Catherine Ho, Teresa Inkster, Emma Hooker

**Affiliations:** Antimicrobial Resistance and Healthcare Associated Infection (ARHAI) Scotland, NHS Scotland Assure, Public Services Delivery Scotland, Glasgow, United Kingdom

**Keywords:** Humidity, Operating room, Operating theatre, Healthcare-associated infections (HAIs), Surgical site infections (SSIs), Infection prevention and control (IPC)

## Abstract

Humidity and temperature ranges for operating theatres are specified in several guidance documents. This rapid review, conducted following an operating theatre humidity deviation incident, examined the evidence underpinning these specifications and found that, although intraoperative humidity is routinely regulated, its role in preventing surgical site infections remains largely speculative. The limited and inconsistent evidence highlights the need for well-designed empirical studies to address this persistent gap in the literature.

## Introduction

Surgical site infections (SSIs) are among the most commonly reported healthcare-associated infections (HAIs). SSIs are often associated with longer postoperative stays, additional surgical procedures, and increased morbidity and mortality [1,2].

It is generally accepted that the causes of SSIs are multi-factorial, and several infection prevention and control interventions are recommended to reduce their incidence. These interventions involve a combination of environment-, equipment-, healthcare worker–, and patient-focused measures. Patient-focused measures include interventions such as nasal decolonisation, pre-operative showering, antiseptic skin preparation, and specific closure methods [2]. Healthcare worker–focused interventions include adherence to aseptic practices, including surgical hand antisepsis and the use of sterile gowns and gloves [2]. Equipment-focused measures pertain to decontamination and sterilisation [3].

Operating theatre or room environment as a potential contributor to surgical safety and optimal outcomes should also be considered. Several guidelines and guidance documents specify that the recommended relative humidity (RH) for the operating room should be between 20% and 60% [[4], [5], [6]], with a maximum of 70% specified in UK guidance [6]. However, these specifications are primarily intended to ensure comfortable conditions for human occupancy, prevent electrostatic discharge, and minimise the risk of mould growth and dust mite proliferation [3,5]. It has also been noted that humidity can affect the shelf life and integrity of some medical supplies, such as electrocardiography electrodes and biological or chemical indicators [3,5,7]. However, these recommendations are based largely on theoretical risk assessments, manufacturer specifications, and expert consensus rather than on experimental evidence or documented incidents.

Some studies have investigated how varying environmental conditions, including humidity, affect bacterial and fungal contamination in operating rooms [8]. However, it remains unclear whether RH influences the risk of SSI. A recent incident involving elevated humidity in a Scottish operating theatre raised safety concerns and prompted a renewed scrutiny of the evidence supporting current guidance recommendations on RH in operating theatres. This may not be an isolated event, given that ageing healthcare estates and warmer summers in Scotland may increase the likelihood of humidity fluctuations above guideline thresholds. A rapid review was conducted by Antimicrobial Resistance and Healthcare Associated Infection (ARHAI) Scotland to summarise findings from studies that examined the relationship between operating theatre humidity and SSIs.

## Methods

A database search was conducted on 21^st^ July, 2025, using a tailored strategy on Ovid MEDLINE and Embase (see Appendix 1) to identify relevant literature published since 2005 that examined the association between operating theatre humidity levels and SSI risk. Additional hand-searching of reference lists and grey literature searches were performed using terms such as ‘humidity’, ‘air’, ‘operating theatre’, ‘surgical suite’, and ‘surgical infection’ within online resources such as the websites of internationally recognised healthcare organisations that produce evidence-based guidance.

Titles and abstracts were screened by a single author, who also conducted the full-text screening. Evidence was critiqued by a single reviewer and extracted for a narrative synthesis. Critical appraisal tools were not used because of the time constraints associated with the incident response.

### Inclusion criteria

Studies were included if they were prospective or retrospective observational studies, case studies, case series, outbreak reports, or systematic reviews with meta-analysis, and published in English. Only studies that explored the risk of SSI associated with operating theatre humidity levels were considered.

### Exclusion criteria

Studies were excluded if they were conference abstracts, narrative reviews without meta-analysis, modelling studies, not published in English, focused on contamination of the surgical field rather than infection risk, or conducted outside surgical or operating theatre settings.

## Results

Two studies were identified: a retrospective cohort study and a prospective longitudinal survey, published in the USA and Spain, respectively [9,10].

The USA retrospective study examined 14,519 surgical cases performed between 2016 and 2020, linking temperature and RH measurements made every 15 min between incision start and closure times. This study included procedures performed in neurosurgery, otolaryngology, urology, gynaecology, and, in general, colorectal, vascular, cardiothoracic, orthopaedic, and plastic surgeries. Ambient temperature and RH in the operating theatres were recorded every 15 min using a wireless sensor throughout the day. The temperature and RH data from the operating rooms were extracted and matched to the surgical procedures by location and the recorded times of incision start and closure. The superficial SSI rate was 1.2% (*N* = 179), with a median intraoperative temperature and RH of 20.1°C and 43%, respectively. Neither low nor high RH was associated with increased superficial SSI risk (odds ratio [OR] = 0.96, 95% confidence interval [CI] = 0.58–1.61; OR = 0.61, 95% CI = 0.33–1.14). Combined temperature–humidity analyses showed no increased risk at extreme values, although procedures performed in low-temperature (<18°C), high-RH (>55.5%) conditions had a lower SSI risk than those performed under medium-range conditions (OR = 0.09, 95% CI = 0.001–0.65, *P* = 0.009) [9].

In contrast, the Spanish prospective study followed 18,910 patients over 1 year and reported an overall SSI incidence of 6.7% (*N* = 1267). This study included procedures performed in neurosurgery and in cardiac, vascular, general, digestive, thoracic, trauma, and orthopaedic surgeries. Temperature and humidity in the operating theatres were measured daily using the Velocicalc Plus tool (model 8386A, TSI Inc.). Mean operating theatre humidity was significantly higher among patients who developed SSI (superficial, deep, and organ/space) than those who did not (54.71% ± 5.055 vs 48.76% ± 3.560, *P* < 0.05). Humidity emerged as one of several significant independent risk factors for superficial SSI (OR = 1.35, 95% CI = 1.28–1.43, *P* < 0.001). Temperature and humidity were measured continuously in the operating room, with mean daily measurements, which may not accurately represent conditions at the exact time of a procedure, used for analysis [10]. In both studies, the follow-up period was 30 days.

## Discussion

The existing literature on the association between RH and the risk of SSI is both limited and contradictory. The retrospective analysis performed within the USA study reported no significant independent association between RH deviations and SSI risk and even suggested a protective effect for certain low-temperature/high-humidity combinations. The Spanish prospective study, however, identified higher mean humidity as a statistically significant independent risk factor for superficial SSI. The contradictory results may stem from differences in how humidity was measured: procedure-specific vs average daily measurement, which makes a direct comparison between the two studies impractical. Other factors, such as differences in patient populations/risk factors, climate, and hospital infrastructure, may limit comparability and impede generalisability to regions with different baseline environmental conditions and microbial ecologies.

In conclusion, although maintaining optimal intraoperative conditions is important for comfort and adherence to aseptic practice, the link between humidity and SSI or other surgical outcomes, such as product and equipment failures that could indirectly influence SSI risk, remains tenuous. Existing concerns about low or high humidity are based largely on theoretical risk assessments, engineering principles, and expert opinion rather than documented incidents or experimental demonstrations. Ultimately, although guidance documents specify acceptable intraoperative humidity ranges, its role in SSI development remains largely speculative. However, when recommended humidity levels are exceeded, a multi-disciplinary team should undertake a risk assessment with regards to continuing procedures and to discuss corrective actions.

The current evidence base consists of theoretical risks and inconsistent observational findings rather than demonstrated clinical consequences. Closing this gap will require well-designed empirical studies to establish if humidity is a modifiable risk factor for SSI. Future studies should attempt to control for various confounders, including surgery duration, baseline contamination risk, patient-specific risk factors, and environmental factors such as operating room design. The use of standardised SSI definitions would also support analysis. Prospective longitudinal studies, designed with consideration of these confounders, across diverse climatic zones and surgical settings will provide valuable insight. It is acknowledged, however, that as SSI development is multi-factorial, further primary studies may only provide evidence of correlation rather than causation. Nevertheless, establishing a more robust evidence base would support the development of guidelines for optimising operating room environments and enhancing patient safety.

## CRediT authorship contribution statement

**Oluwadamilola Fasan:** Writing – original draft, Methodology, Investigation, Conceptualisation. **Ogheneochuko Andrew Saba:** Writing – original draft, Methodology, Investigation. **Lorna Gordon:** Writing – review & editing, Supervision, Project administration, Conceptualisation. **Catherine Ho:** Writing – review & editing, Methodology, Investigation. **Teresa Inkster:** Writing – review & editing. **Emma Hooker:** Writing – review & editing, Supervision, Conceptualisation.

## Ethical approval

Not required.

## Funding sources

None.

## Conflict of interest statement

None declared.

## Figures and Tables

**Table:** 

Line	Search term	Result
1	Operating theatre∗ or operating room∗ or surgical theatre∗ or surgical suite∗ or surgical room∗ or procedur∗ room∗ or surgery or surgeries	8,250,524
2	Healthcare associated infection∗ or surgical site infection∗ or prosthes∗ infection or surgical infection∗ or nosocomial infection∗ or post-operative infection∗ or post surgical infection∗ or healthcare acquired infection∗ or hospital associated infection∗ or hospital acquired infection∗ or HAI	201,005
3	Humid∗ or (air adj3 moisture) or (environment∗ ajd3 moisture)	173,725
4	1 and 2 and 3	137
5	Limit 4 to human studies, 2005-current, English language.	109
6	Limit to yr=2005 – current	108
7	Remove duplicates	89
